# Efficacy of a nutraceutical combination on lipid metabolism in patients with metabolic syndrome: a multicenter, double blind, randomized, placebo controlled trial

**DOI:** 10.1186/s12944-019-1002-y

**Published:** 2019-03-18

**Authors:** Ferruccio Galletti, Valeria Fazio, Marco Gentile, Giuseppe Schillaci, Giacomo Pucci, Francesca Battista, Valentina Mercurio, Giorgio Bosso, Domenico Bonaduce, Nadia Brambilla, Cristina Vitalini, Massimo D’Amato, Giampaolo Giacovelli

**Affiliations:** 1 Department of Clinical Medicine and Surgery, ESH Excellence Centre of Hypertension, “Federico II” University of Naples Medical School, Naples, Italy; 2 Unit of Internal Medicine, Terni University Hospital, Department of Medicine, University of Perugia, Perugia, Italy; 3 Department of Translational Medical Sciences, “Federico II” University of Naples Medical School, Naples, Italy; 4 Rottapharm Biotech, Monza, Italy

## Abstract

**Background:**

Nutraceuticals represent a new therapeutic frontier in the treatment of metabolic syndrom (MetS) and related cardiovascular risk factors. The aim of this study was to evaluate the potential beneficial effects of Armolipid Plus (AP) (berberine 500 mg, red yest rice, monacolin K 3 mg and policosanol 10 mg) on insulin resistance, lipid profile, particularly on small and dense LDL cholesterol (sdLDL-C), representing the most atherogenic components, as well as its effects on high sensitivity C-reactive protein, a notable marker of cardiovascular risk, blood pressure and cardiac remodeling in subjects affected by MetS, with left ventricular hypertrophy.

**Methods:**

The study was a prospective, multi-center, randomized, double blind, placebo-controlled trial. One hundred and fifty eight patients, aged between 28 and 76 years old, were enrolled and randomized to receive either one tablet of AP or placebo (PL) once daily for 24 weeks. Anthropometric and vital parameters, total cholesterol (tot-C), low-density lipoprotein cholesterol (LDL-C), high density lipoprotein cholesterol (HDL-C), triglyceridemia (TG), non-HDL cholesterol (NHDL-C) and sdLDL-C were evaluated.

**Results:**

After 24 weeks of treatment, the analysis performed on 141 subjects (71 in AP arm and 70 in PL arm), showed a significant improvement of lipid profile in the AP group, with reduction in tot-C (− 13.2 mg/dl), LDL-C (− 13.9 mg/dl) and NHDL-C (− 15.3 mg/dl) and increase in HDL-C (+ 2.0 mg/dl). These changes were equally significant compared with placebo (tot-C: AP − 13.2 mg/dL vs PL + 2.7 mg/dL, *p* < 0.01; LDL-C: AP -13.9 mg/dl vs PL + 1.5 mg/dl, p < 0.01; NHDL-C: AP -15.3 mg/dl vs PL + 2.8 mg/dl, p < 0.01), Although no significant difference was observed between the two arms in the reduction of HDL-C nevertheless it increased significantly in the AP group (AP + 2 mg/dL *p* < 0.05, PL 0.13 mg/dL).

**Conclusion:**

The results of this study, applicable to a specific local population show that, in a population of subjects affected by MetS, treatment with AP improves the lipid profile and the most atherogenic factors, thus suggesting a reduction in the risk of development and progression of atherosclerosis, particularly in subjects with high atherogenic risk, due to the presence of sdLDL-C.

## Introduction

Metabolic syndrome (MetS) is a common clinical condition in the western world, associated with an increased risk for cardiovascular disease (CVD) [1, 2].

This condition is characterized by visceral obesity, high blood pressure values and insulin resistance (IR), which promotes an atherogenic lipid profile, with raised triglycerides (TG), elevated very low density lipoprotein cholesterol (VLDL-C) and decreased high density lipoprotein cholesterol (HDL-C), which translates into an increase in non-HDL cholesterol (NHDL-C).

Insulin resistance is a state in which physiological amount of insulin has a reduced effect on post-prandial glicemic control with an inadequate insulin suppression during the night period. Initially it is counteracted by increasing the release of insulin to maintain normal glucose serum value. Over time, this compensatory mechanism tends to become inadequate, so patients develop postprandial and then fasting hyperglycemia. It also reduces glucose uptake, causing a reduction in muscle glycogen stores, promotes hepatic gluconeogenesis and increases sympathetic tone. It was demonstrate that hyperinsulinemia, in non diabetic patients, is associated with raised incidence of cardiovascular events, independently of metabolic lipid profile [1, 2].

It is well known that hypercholesterolemia is an independent risk factor for CVD and particularly low-density lipoprotein cholesterol (LDL-C) levels above the upper normal limit caused an increased cardiovascular risk. Using electrophoresis can be identified 7 subfractions of LDL-C, relative to decreasing size and increasing density. The subfractions 3 to 7, commonly identified as small-dense LDL-C (sdLDL-C), are proven to be more atherogenic than larger LDL-C particles, due to their longer persistence in circulation, their greater susceptibility to oxidation and glycation and enhanced affinity for proteoglycans in the arterial wall [3–10]. It was observed that elevated sdLDL-C plasma concentration tends to correlate with high plasma TG and low HDL-C levels, which are key features of MetS. Moreover, the increase in sdLDL-C level seems to be directly related to the numbers of the components of MetS, so it could represent a marker for diagnosis and severity of this syndrome [11–16]. Clinical studies demonstrated that elevated sdLDL-C plasma concentration is significantly associated with increased risk in CVD [17–21].

The Adult Treatment Panel III report has identified MetS as a secondary target of therapy in the management of CVD, in addition to LDL-C lowering therapy [22–25].

The wide phenotypic heterogeneity of MetS and its complex pathogenesis, make difficult to identify a single therapeutic target. Current therapeutic approach of the syndrome is based on stable lifestyle changes, and often requires a complex multi-drug regimen, addressing the components of the syndrome individually. This translates into high costs, poor compliance and few results, with an increased risk of side effects.

Hypolipidemic drugs, today available, have proven efficacy in reduction of LDL-C and also sdLDL-C. Nevertheless, they have been shown to have relevant interactions with other commonly used drugs and not negligible side effects.

Non-pharmacological options for dyslipidemia treatment could be a good alternative, improving patient compliance [26].

Recent studies demonstrate the efficacy of nutraceutical combinations in reduction of lipid plasma values, without increasing in CPK level, myalgia or myopathy, which makes them good candidates for cholesterol-lowering therapy in patients intolerant to common treatments [27–29]. Moreover, this therapy has proved also safe in children affected by heterozygous familial hypercholesterolemia [30].

Aim of this study was to investigate the effects of a nutraceutical combination on insulin resistance, lipid metabolism, and on LDL-C subtypes, in patients with MetS and left ventricular hypertrophy. The nutraceutical combination used in this study consisted of a single tablet containing berberine (BRB 500 mg), red yest rice (RYR) (monacolin K 3 mg) and policosanol 10 mg (Armolipid Plus®, AP, Rottapharm Madaus, Italy).

## Materials and methods

### Study design

The study was a prospective, multi-center, randomized, double blind, placebo-controlled trial, consisting of a screening visit and a 24 week treatment period. It was conducted at three centers, including two at the University of Medicine and Surgery of Naples “Federico II” and one at the Terni University Hospital. The Institutional Ethics Committee of each site approved the study protocol before initiating any trial related activity (Università degli Studi di Napoli Federico II: protocol number 13/12 for center 1 and 28/12 for center 3; Aziende Sanitarie Umbria: protocol number 2026/12), and written informed consent was obtained from each patient. The study was conducted in accordance with the principles of the Declaration of Helsinki.

ClinicalTrials.gov Identifier: NCT02295176.

### Patients and treatment

Study participants were recruited between April 2013 and July 2014.

Eligibility criteria were: (1) age between 18 and 70 years; (2) diagnosis of MetS, defined as the presence of a waist circumference ≥ 94 cm (male), ≥ 80 cm (female), associated with at least two of the following: systolic blood pressure (SBP) ≥ 130 mmHg or diastolic blood pressure (DBP) ≥ 85 mmHg or need for antihypertensive therapy; fasting glucose (FG) ≥ 100 mg/dL; HDL-C < 40 mg/dL (male), < 50 mg/dL (female); TG ≥ 150 mg/dL or need for lipid-lowering therapy; (3) left ventricular mass (LVM) > 48 g/m^2,7^ (male) or > 44 g/m^2,7^ (female) and (4) ability to understand and give informed consent to clinical experimentation.

Exclusion criteria were: (1) proven intolerance to any component of the nutraceutical compound; (2) pregnancy or breastfeeding; (3) treatment with hypoglycemic agents and/or glycated hemoglobin (HbA1c) ≥ 6.5%; (4) moderate to severe liver dysfunction (Child B-C); (5) abnormal renal function (eGFR < 30 mL/min/1.73 m^2^); (6) serum triglycerides > 500 mg/dL; (7) severe obesity (body mass index (BMI) > 35 Kg/m^2^); (8) a history or current symptoms of heart failure; (9) left ventricular systolic dysfunction (left ventricular ejection fraction (LVEF) ≤ 40%); (10) hypertrophic cardiomyopathy; (11) heart valve stenosis; (12) previous myocardial infarction; (13) pacemaker-induced ventricular rhythm; (14) moderate to severe heart valve regurgitation; (15) uncontrolled hypertension despite optimum therapy (PAS > 140 mmHg o PAD > 90 mmHg). Patients with concomitant disease were included, provided their clinical conditions and treatments had been stable during the previous three months and have not had major clinical events.

One hundred and fifty eight patients, meeting the eligibility criteria, were enrolled in the study. Patients were randomized to receive either one tablet of Armolipid Plus.

(MEDA-Rottapharm SpA; 1 tablet/day, containing 200 mg of RYR [equivalent to 3 mg of monacolin K], 500 mg of berberine, 10 mg of policosanols, 0.2 mg of folic acid, 2.0 mg of coenzyme Q10 and 0.5 mg of astaxanthin; RYR contained in Armolipid Plus was citrinine and aflatoxins free) or placebo (1 tablet/ day, identical in taste and appearance to the Armolipid Plus tablet but containing microcrystalline cellulose, iron oxide brown 70, Compritol E ATO [Gattefoss_e Saint-Priest, Lyon, France] and magnesium stearate). Randomization and blinding were provided by Rottapharm Madaus SpA (Monza, Italy), which also funded the study. The randomization was performed 1:1 ratio according to a computer-generated randomization list, containing the randomization codes to assign progressively to the patients and used for treatment dispensing. The study staff and the investigators, as well as all the patients, were blinded to the group assignment. The randomization codes were kept in a sealed envelope, which was not opened until study completion and data analysis. In the placebo group 44 patients were being treated with statins. Similarly, in the AP group, 44 patients were being treated with statins.

### Outcomes

The primary endpoints were to confirm metabolic effects of AP on insulin resistance (IR), in patients with MetS and left ventricular hypertrophy and to evaluate the effects of treatment on lipid profile, particularly on small and dense LDL cholesterol (sdLDL-C), representing the most atherogenic components. Secondary endpoints were its effects on cardiac remodeling, blood pressure, and C-reactive protein Hs (CRP-Hs), a notable marker of cardiovascular risk. This article will address only the metabolic part of the clinical trial.

### Clinical and strumental measurements

All patient underwent an initial screening which included anamnesis, physical examination and evaluation of the inclusion/exclusion criteria. Study assessments were performed at baseline and after 24 wk. of treatment. At baseline, for each patient, were recorded medical history, physical examination with assessment of anthropometric parameters and vital signs. Patient’s stature was calculated with the subject standing upright, with the back of the skull, shoulders and lower limbs in contact with the altimeter; the weight was calculated with the subject wearing just light clothes and the waist circumference was calculated with an anelastic meter, in contact with the skin, positioned at the major extension of the abdomen. The measurement was performed at the end of a normal exhalation. Routine blood tests were also performed, including lipids, fasting glucose and insulin level. Blood samples for the assessment of study parameters have been analyzed from coordinating center. LDL particles were separated by Lipoprint System (Quantimetrix Inc., Redondo Beach, California). Were obtained seven LDL subfractions, decreasing size and increasing density, and electrophoretic mobility. Mean LDL particle diameter was calculated on the basis of the different areas under the curve of the 7 LDL species with different electrophoretic mobility. The proportion of sd-LDL particles (subfractions 3–7) to the whole LDL area (subfractions 1–7) was also calculated in our sample (LDL score). LDL score, has been shown to be significantly related to coronary heart disease (CHD) in multivariate analysis [16]. Hs-CRP were measured with turbidimetric assay, using automated methods (Pentra 400, Horiba Medical, USA). The error of the method was evaluated daily by analyzing a plasma pool and was 0.5%.

It was recorded a 12-lead ECG at rest and performed basal echocardiography. All recordings of the echocardiographic exams were evaluated, blinded, by one single operator of the coordinating center. The evaluation of LVM was performed according to the recommendations of the American Society of Echocardiography [31]. Patients were randomized to receive the treatment. After 4 wk. of treatment it was planned a phone call to verify treatment adherence, recording any changes in concomitant medications and adverse events. After 12 wk. the patients underwent a new clinical visit and blood tests to monitoring safety parameters. Concomitant medications and adverse events were monitored throughout the study.

### Statistical analysis

Statistical analyses were performed using SAS software (version 9.3; SAS Institute, Cary, NC, USA). Descriptive results were expressed as mean ± standard deviation (SD) or percentages, according to the type of variable. Changes in efficacy parameters from baseline to 24 wk. were compared between treatment groups by means of a one-way analysis of variance (ANOVA) with treatment as factor. In addition, to take into account the statins use when assessing the treatment effect, an ANOVA with treatment and statins use (yes/no) as factors was performed. Comparisons within treatment group between baseline and value at wk. 24 were carried out by means of the paired t-test. Significance for all analyses was set at *P <* 0.05.

## Results

**Fig. 1:**
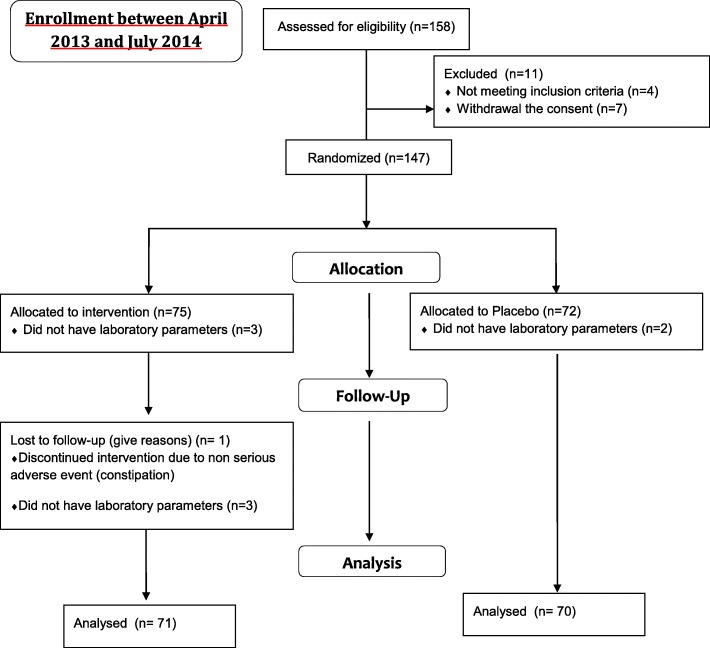
Study flow diagram

One hundred fifty eight patients, aged between 28 and 76 years, meeting the eligibility criteria of our study, were enrolled. One hundred forty one patients (71 in the AP arm and 70 in the PL arm) had laboratory parameters analyzed from coordinating center. Of the remaining 17, 5 patients did not have laboratory parameters analyzed from coordinating center, whereas 12 patients prematurely discontinued the study (4 because of protocol violation of inclusion/exclusion criteria, 7 due to withdrawal of consent and 1 patient, in AP group, due to non-serious adverse events related to the treatment) (Fig. 1).

Patients’ clinical characteristics are shown in Table 1. At baseline, the 2 groups were comparable in age, sex, concomitant hypolipidemic medications, anthropometric parameters, and lipid levels. In the placebo group 44 patients were being treated with statins. Similarly, in the AP group, 44 patients were being treated with statins.

**Table 1:** Baseline characteristics, mean ± SD or n (%)

	ARMP (*n* = 71)	Placebo (*n* = 70)
Age (years)	55.6 ± 8.9	55.6 ± 9.3
Age groups		
< 65 years	61 (85.9%)	56 (80.0%)
≥ 65 years	10 (14.1%)	14 (20.0%)
Sex		
Males	42 (59.2%)	37 (52.9%)
Females	29 (40.8%)	33 (47.1%)
Smoking		
Yes	17 (23.9%)	15 (21.4%)
No	54 (76.1%)	55 (78.6%)
Weight (kg)	80.6 ± 12.6	80.7 ± 12.1
Height (cm)	165.5 ± 8.9	166.1 ± 8.0
BMI (kg/m^2^)	29.4 ± 3.6	29.2 ± 3.5
Waist circumference (cm)	100.8 ± 9.3	100.3 ± 8.7
SBP (mmHg)	130.6 ± 10.5	131.4 ± 10.6
DBP (mmHg)	80.7 ± 8.1	81.6 ± 8.0
Pulse rate (beats/min)	65.3 ± 9.5	68.3 ± 10.5
Ongoing-lipid lowering treatment		
Yes	41 (57.8%)	41 (58.6%)
No	30 (42.2%)	29 (41.4%)
Tot-C (mg/dL)	224.3 ± 44.7	218.4 ± 38.2
LDL-C (mg/dL)	132.9 ± 36.5	128.4 ± 28.6
NHDL-C (mg/dL)	173.6 ± 41.8	168.0 ± 36.3
HDL-C (mg/dL)	50.7 ± 11.9	50.4 ± 12.1
TG (mg/dL)	151.3 ± 82.5	159.6 ± 86.6
LDL score	6.6 ± 7.1	6.8 ± 8.2
HbA1c (%)	5.5 ± 0.4	5.5 ± 0.4
Glucose (mg/dL)	103.9 ± 14.5	105.7 ± 17.9
HOMA-IR	4.1 ± 3.2	4.2 ± 2.4
Insulin (μU/ml)	15.7 ± 11.6	16.3 ± 9.0
CRP-Hs (Mg/L)	1.85 ± 2.34	1.35 ± 1.01
Stratum (number of subject)		
Missing LDL score	2	1
LDL score = 0	15	19
LDL score > 0	54	50

After 24 wk. of treatment, the comparison of absolute changes from baseline between the 2 groups showed no significant variations for fasting plasma glucose and insulin, just as homeostasis model assessment of insulin resistant (HOMA-IR) index, while a significant improvement in lipid profile was detected in the AP arm (Tab.2) with decrease in total cholesterol (Tot-C) (AP = − 11 mg/dl vs PL:+ 3, *p* < 0.01), decrease in LDL-C (AP = − 14 mg/Dl vs PL = + 2, p < 0.01), in NHDL-C (AP = -15 mg/Dl vs PL = + 3, p < 0.01) while no significant difference was observed between the two arms in the reduction of TG (AP = − 9 mg/dL, PL = + 4, *P* > 0.05) (Table 2).

**Table 2:** Changes in lipid profile after 24 weeks of therapy

	AP (*n* = 71)		PL (*n* = 70)	
	Baseline	24 weeks	Baseline	24 weeks
Tot-C (mg/dL)	224 ± 45	211 ± 44*	218 ± 38	221 ± 40
LDL-C (mg/dL)	133 ± 36	119 ± 33*	128 ± 29	130 ± 32
NHDL-C (mg/dL)	174 ± 42	158 ± 41*	168 ± 36	171 ± 39
HDL-C (mg/dL)	51 ± 11	53 ± 13*	50 ± 12	50 ± 10
TG (mg/dL)	151 ± 82	142 ± 93^§^	160 ± 87	164 ± 100

* = *p* < 0.01 ARM vs Placebo. ^§^ = *p* < 0.05 24 weeks vs Baseline

Although no significant difference was observed between the two arms in the reduction of HDL-C nevertheless it increased significantly in the AP group (AP = + 2 mg/dL *p* < 0.05, PL = 0,) (Table 2).

**Fig. 2:**
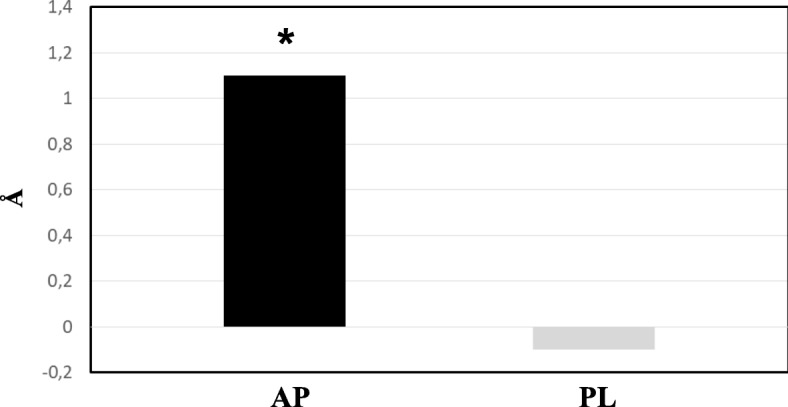
Changes in sdLDL-C size at wk. 24. * = *p* < 0.05 24 wks vs Baseline

In addition, in a subgroup of 104 patients with presence of sdLDL-C (54 in the AP group and 50 in PL group) (Table 1), only subjects who received AP had a significant improvement in sdLDL-C size at wk. 24 (from 267 ± 3 to 268 ± 3 Å, *P <* 0.05 for within group comparison) (Fig. 2). Moreover, in this subgroup persist more clearly the differences between the two treatment arms.

**Fig. 3:**
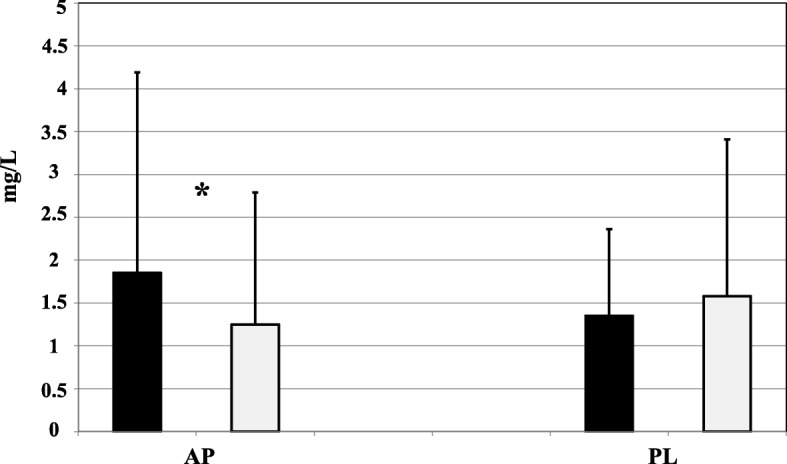
CRP-Hs levels after 24 weeks of treatment.  = AP,  = PL Treatment. * = *p* < 0.05 24 wks vs Baseline

Finally, we observed a significant improvement in CRP-Hs in AP group compared with PL (from 1.85 ± 2.34 to 1.25 ± 1.54 mg/L vs from 1.35 ± 1.01 to 1.58 ± 1.83 mg/L, *P* < 0.05) (Fig. 3).

No significant variations were observed in body weight, waist circumference and blood pressure, both within-group and between-group.

No changes in renal and hepatic parameters were observed throughout the study period. AP was generally well tolerated and no serious adverse event related to sperimental treatment occurred.

## Discussion

Our study does not confirm AP effects on IR, whereas confirmed the efficacy and safety of AP in improving lipid metabolism, in a local population of subjects suffering from MetS. in patients with MetS. After 24 weeks of treatment we observed a significant reduction in tot-C, NHDL-C and LDL-C, with increase in sdLDL-C size.

LDL-C includes particles varying in density, composition, size and electrical charge. In relation to the LDL-C subgroup, isolated from plasma, it is possible to distinguish two phenotypes: pattern A characterized by a predominance of large buoyant LDL-C (lbLDL-C) and pattern B with a predominance of sdLDL-C, traditionally involved in cardiovascular risk, because of their high atherogenic power. These particles show a lower affinity for LDL-C receptor and remain in circulation longer. Moreover, their composition makes them more prone to chemical modification. Numerous studies have investigated the influence of sdLDL-C on CVD risk [18–22]. It was observed a direct relation between sdLDL-C and TG plasma levels and a reverse relation between sdLDL-C and HDL-C plasma levels. The exact mechanism underlying the association between TG and sdLDL-C concentrations it is still unknown. It has been speculated that excess adiposity, especially in the presence of diabetes or insulin resistance, leads to increase in free fatty acid (FFA) production by adipocytes, which promote an increased hepatic triglyceride synthesis. This develops an increased synthesis and secretion of VLDL-C by the liver. Through the action of cholesterol ester transfer protein; an appreciable amount of triglyceride in VLDL-C may be exchanged for cholesterol ester in plasma LDL-C. These triglyceride-enriched-LDL-C particles may be transformed into sdLDL-C particles, by lipase-mediated triglyceride hydrolysis, in the liver. In a recent paper Barrios at al. have evaluated the effect of Armolipid plus in individuals with mild to moderate dyslipidemia treated for 6–48 weeks. Authors reported asignificant reductions in Total Cholesterol (11–21%) and in LDL-C (15–31%) levels but sdLDL-C were not evaluated [32]. This study was the first to evaluate the nutraceutical effects of berberine on sdLDL in a population with MetS. A supposed mechanism of the lipid-lowering effects of Armolipid Plus is due to the synergic effect of RYR, berberin, policosanol, astaxanthin, coenzyme Q10 and folic acid. RYR extract, in particularly monacolin K, has an inhibitory activity on HMG-CoA reductase, in addiction Berberine has been suggested to lead to an increased expression and half-life of the LDL receptor (LDLR) on the surface of hepatocytes [33]. After the treatment period, we found an increase in HDL-C level in AP arm, compared with PL arm, which is commonly considered as a protective agent against cardiovascular disease.

This study, for the first time, evaluated the nutraceutical effects of BRB on sdLDL-C in a population with MetS. After the treatment period, we found a significant improvement in sdLDL-C size associated with an increase in HDL-C level in AP arm, compared with PL arm, which is commonly considered as a protective agent against cardiovascular disease.

Finally, patients in the active arm also showed an improvement in NHDL-C levels, classically elevated in MetS, which seems to be notable because of its contribution to cardiovascular risk and endothelium damages, also confirmed by the decrement in CRP-Hs values found during AP treatment.

It is interestingly to note that our data confirm that the therapeutic effect of AP is evident also in those patients who were already under treatment with statins [32].

From a “pharmacodynamic” standpoint, some of these results can be due to the effects of BRB on lipid metabolism. BRB increase expression of LDL receptor (LDLR) on hepatocytes membrane, promoting its gene transcription, by inducing stabilization of LDLR-mRNA, and inhibiting its lysosomial degradation PCSK9 mediated [33]. This mechanism is independent of intracellular cholesterol levels and could counteracts the inducing effect of statins on PCSK9, that results in a counterproductive increase in LDLC plasma levels, which tends to reduce the lipid-lowering effect of the drug. Pharmacological and nutraceutical combination may translate into a synergistic efficacy. BRB also is able to inactivate acetyl CoA carbossilase, via adenosine mono-phosphate kinase, a key enzyme involved in fatty acid synthesis, leading to an increase in fatty acid oxidation, decrease in fatty acid synthesis and TG synthesis inhibition [33].

There are some limitations that we need to take into account, such as short length of the study follow-up. Certainly the other important limitation of this study is the presence of cholesterol lowering therapy; however, this choice was determined by ethical reasons, which led us to choose an “on-top-of” design. In addition, we hypothesize that the lack of effect of ARM on insulin sensitivity might be due to statin treatment, given its well-known positive effect on insulin sensitivity.

## Conclusion

This is the first study which evaluated the nutraceutical effect, of compound such as BRB, on sdLDL-C in a population with high CVD risk, as patients affected by MetS. Our results have shown that, in a population of subjects suffering from MetS, treatment with AP is effective and safe to improve the lipid profile and the more atherogenic factors, also in patients already under treatment with statins, thus reducing the risk of development and progression of atherosclerosis, particularly in individuals with high risk of CVD for the presence of atherogenic sdLDL-C.

The safety profile of the combination therapy support its use in patients that do not tolerate statins or do not achieve therapeutic goals with single therapy.

Further studies with a long-term follow-up are needed to confirm these promising results and evaluate their effects on morbidity and mortality from cardiovascular disease.

## Abbreviations

AP: Armolipid Plus
CVD: cardiovascular disease
HDL-C: high density lipoprotein cholesterol
IR: insulin resistance
LDL-C: low-density lipoprotein cholesterol
MetS: metabolic syndrom
NHDL-C: non-HDL cholesterol
PL: placebo
sdLDL-C: small and dense LDL cholesterol
TG: triglyceride
Tot-C: total cholesterol
VLDL-C: very low density lipoprotein cholesterol
